# Key genes and regulatory networks involved in the initiation, progression and invasion of colorectal cancer

**DOI:** 10.4155/fsoa-2017-0108

**Published:** 2018-01-24

**Authors:** Matin Asghari, Mohammad Foad Abazari, Hanieh Bokharaei, Maryam Nouri Aleagha, Vahdat Poortahmasebi, Hassan Askari, Sepehr Torabinejad, Abbas Ardalan, Navid Negaresh, Atousa Ataei, Parisa Pazooki, Mansour Poorebrahim

**Affiliations:** 1 Department of Molecular Biotechnology, Cell Science Research Center, Royan Institute of Biotechnology, ACECR, Isfahan, Iran; 2 Department of Genetics, Islamic Azad University, Tehran Medical Branch, Tehran, Iran; 3 Department of Genetics, Faculty of Basic Sciences, Science & Research Branch, Azad University, Tehran, Iran; 4 Hepatitis B Molecular Laboratory, Department of Virology, School of Public Health, Tehran University of Medical Sciences, Tehran, Iran; 5 Department of Physiology, Faculty of Medicine, Tehran University of Medical Sciences, Tehran, Iran; 6 Department of Biology, Faculty of Sciences, Arak University, Arak, Iran; 7 Department of Medicine, Faculty of Medicine, Qom Branch, Islamic Azad University, Qom, Iran; 8 Institute of Fundamental Medicine & Biology, Kazan Federal University, Kazan, Russia; 9 Department of biological sciences, Tehran north branch, Islamic Azad university, Tehran, Iran; 10 Department of Medical Biotechnology, School of Advanced Technologies in Medicine, Tehran University of Medical Sciences, Tehran, Iran

**Keywords:** biomarkers, CRC stages, hub genes, PPI networks, signaling pathways

## Abstract

**Aim:**

Until now, identification of drug targets for treatment of patients with specific stages of colorectal cancer (CRC) has remained a challenging field of research. Herein, we aimed to identify the key genes and regulatory networks involved in each stage of CRC.

**Results:**

The results of gene expression profiles were integrated with protein–protein interaction networks, and topologically analyzed. The most important regulatory genes (e.g., *CDK1*, *UBC*, *ESR1* and *ATXN1*) and signaling pathways (e.g., Wnt, MAPK and JAK-STAT) in CRC initiation, progression and metastasis were identified. *In vitro* analysis confirmed some *in silico* findings.

**Conclusion:**

Our study introduces functional hub genes, subnetworks, prioritizes signaling pathways and novel biomarkers in CRC that may guide further development of targeted therapy programs.

## Lay abstract

A comprehensive view of the molecular mechanisms involved in colorectal cancer (CRC) pathogenesis has been demonstrated. Cell cycle and signal transduction genes were contributors to CRC initiation and progression. In contrast to stage IV, the stages I–III of CRC shared a notable number of common genes suggesting their close gene signatures. The efficacy of these predictions has been validated using two colon cancer cell lines *in vitro*.

## Graphical abstract

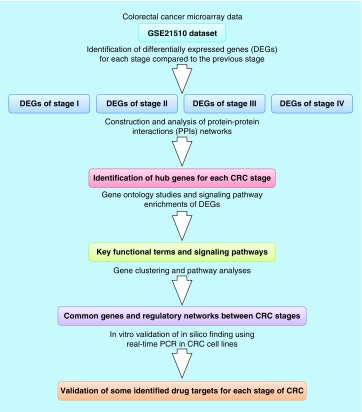

Colorectal cancer (CRC) is known as the third leading cause of cancer mortality worldwide, propagating by the acquisition of several genetic alternations influencing the expression and function of target genes. Adenomatous Polyposis Coli and *TP53* are the most commonly mutated genes in familial/inherited and sporadic CRC [1]. CRC metastasis is a common phenomenon and the major lethal cause of CRC cases, as approximately 50% of CRC patients die within 5 years due to extensive metastatic stage [2]. So far, however, there has been little information about the molecular mechanisms underlying the ability of CRC cells to become invasive. Numerous studies have proved that the transition of CRC cells from early stages to late stages (metastasis) is associated with a significant change in the cellular gene expression profile [3]. These genetic alterations have led to the emergence of the novel strategies for personalized medicine programs [4]. As a result, Zhao *et al*. found an apparent difference between protein expression profile of nonmetastatic and metastatic CRC cell lines. They concluded that overexpression of *HSP27* might play a key role in the acquisition of invasion and migratory properties by CRC cells [5]. Furthermore, Tan *et al*. introduced *STMN1* gene as a crucial player in CRC cell migration and prognostic marker through a comparative proteome analysis of primary CRC cell lines HCT-116 and its metastatic derivative E1 [6]. Most recently, we have identified HOXB family as the most significant functional modules in metastatic CRC samples using a network biology approach. Consequently, we proposed a rationally designed anti-*HOXB7* peptide for preventing CRC metastasis [7]. Despite, these valuable studies, the benign-to-malignant transition of CRC is a complicated multistep process and needs to be further elucidated in greater details.

Nowadays, computational systems biology has become a focus of many researchers to decipher complex biological systems through integrating the experimental and computational approaches [8]. Among the currently available methods, reconstruction of protein–protein interaction (PPI) networks are widely used for identification of key genes and functional modules involved in the cancer initiation and progression [9,10]. There are several network-based methodologies for modeling of biological systems that result in an identification of novel biomarkers in the human cancers and complex diseases [11]. To the best of our knowledge, no attempt has yet been made to monitor the dynamic changes of regulatory networks from normal to distant metastasis of the patients with CRC. Accordingly, no effective biomarkers have been introduced for detecting the early events of CRC metastasis. Hence, we assessed the gene expression changes of normal versus stage I (N-S_I_), stage I versus stage II (S_I_-S_II_), stage II versus stage III (S_II_-S_III_) and stage III versus stage IV (S_III_-S_IV_) of CRC cells to propose a biological model for CRC initiation, progression and invasion to other tissues. Additionally, the overlapping mechanisms between four studied CRC stages were elaborated in more details The observations uncovered that several common genes and subnetworks seem to be associated with CRC initiation and metastasis. These findings would help to gain insights into the major contributors involved in the regulation of CRC initiation, progression and invasion. Our study also provides novel potential biomarkers which can be considered to be therapeutic candidates for designing novel drugs with anti-CRC activity.

## Materials & methods

### Microarray gene expression data

A gene expression dataset (Gene Expression Omnibus [GEO] accession number: GSE21510) consisting of total 148 microarray samples were chosen from (GEO; www.ncbi.nlm.nih.gov/geo/). The samples had been accumulated from LCM and homogenized tissues of patients with colorectal cancer. Subsequently, the samples separately normalized using robust multiarray average method under R 2.6.2 statistical software with affy package from BioConductor. There was only one sample for stage 0, therefore, we excluded this stage from the study. Differentially expressed genes (DEGs) were determined by using the GEO2R tool [12]. Fold change > 1 or < −1 and p-value < 0.05 are the thresholds used in DEGs determination.

### PPI networks

A comprehensive PPI was constructed for each stage using BisoGenet plugin of Cytoscape [13]. This plugin is a multitier application that builds the PPI based on the biomolecular relationships data curated from several PPI databases, including Database of Interacting Proteins, Biological General Repository for Interaction Datasets, Human Protein Reference Database, Biomolecular Interaction Network Database, Molecular INTeraction database (MINT) and IntAct [14–19]. In parallel, an intensive literature search was conducted to retrieve new PPIs from the data of most recent studies. The PPI networks were visualized and analyzed by Cytoscape and FunRich packages [20].

### Identification of hub genes

Topological properties of the PPI networks were interpreted to detect the paramount functional hub genes. We used Network Analyzer, a network analysis plug-in of Cytoscape, to calculate the different topological parameters of resulted PPI networks. To this end, nine measures including Number of Undirected Edges, Degree, Betweenness Centrality, Clustering Coefficient, Closeness Centrality, Eccentricity, Neighborhood Connectivity, Topological Coefficient and Average Shortest Path Length were calculated for each PPI network.

### Gene ontology (GO) & signaling pathway enrichment

Gene ontology (GO) studies were carried out utilizing Biological Networks Gene Ontology tool, a flexible and extendable Cytoscape's plugin [21]. This plugin finds GO terms over-represented in the given biological networks. Enrichr server was used as an alternative tool for validating the results of Biological Networks Gene Ontology tool [22]. A p-value less than 0.05 was chosen as the cut-off criterion for statistically over-represented GO terms. A signaling pathway enrichment was performed using SPEED web tool to identify the paramount signaling pathways underlying CRC initiation and invasion [23]. This server is a signaling pathway annotation tool that performs pathway enrichments based on the data obtained from previous pathway perturbation experiments.In parallel, the signaling pathways corresponding to the DEGs were also checked with the Kyoto Encyclopedia of Genes and Genomes [24]. This database consists of throughput information about genomic, cellular pathways and chemical compounds.

### Construction of DEG-GO network for S_IV_ of CRC

After determining DEGs of stage IV gene expression profile compared with the normal samples, a DEG-GO network was constructed to distinguish the major regulators of significantly over-represented GO terms. First, the up-regulated DEGs were separated and their biological roles were determined using DAVID server to understand the activated molecular mechanisms and signaling pathways in metastatic CRC cells compared with their adjacent normal tissue. The GO terms with p-value < 0.05 containing at least ten genes were considered to be significantly over-represented categories. Finally, a DEG-GO network was constructed and topological analysis of the network was performed using out-degree (the number of outgoing edges) and in-degree (the number of incoming edges) measures.

### Identification of common regulatory networks between CRC stages

Stages 1–4 of CRC may share several common genes or modules and identification of these similarities can be helpful in CRC therapy programs. To this end, all DEGs obtained from the comparison of four stages with their adjacent normal tissues (with fold change >1 and <−1 and p-value < 0.05) were clustered using Venny 2.1 web tool [25]. The main subnetwork for both common up and down-regulated DEGs were separately extracted and interpreted. In parallel, GO analysis of common DEGs was conducted to understand the regulatory functions of these genes.

### Cell culture, RNA extraction & real-time PCR

In order to validate the *in silico* findings, the expression level *ATXN1* and *CDK1*, identified as hub genes in S_IV_ of CRC, was quantified in human metastatic colon carcinoma cell line (SW620 cells) compared with NCM460, a normal human colon mucosal epithelial cell line. Briefly, SW620 and NCM460 cell lines were seeded in Dulbecco's modified Eagle's medium supplemented with 10% fetal bovine serum and 1% penicillin/streptomycin. Total RNA was extracted from SW620 and NCM460 cell lines (15–30 mg) using the miRNeasy Kit according to the manufacturer's instructions (Qiagen, USA). Subsequently, cDNA synthesis was performed utilizing a commercial cDNA synthesis kit (2-step RT-PCR kit, Vivantis, USA). The standard SYBR green-based quantitative real-time PCR (qRT–PCR) analyses were carried out in Real-time PCR Detection System ABI 7300 (Applied Biosystems, USA). Primer sequences of *ATXN1*, *CDK1* and *GAPDH*, as internal control, are as follows: *ATXN1*-F (5′ CCCACCTTTCCCCTGCTGCG-3′), *ATXN1*-R (5′-GGTTCCTCCCCCGGGTCTCC-3′), *CDK1*-F (5′-GGAAACCAGGAAGCCTAGCA-3′), *CDK1*-R (5′-TGATTCAGTGCCATTTTGCC-3′), *GAPDH*-F (5′-TCCACCACCCTGTTGCTGTAG-3′) and *GAPDH*-R (5′-GGTTCCTCCCCCGGGTCTCC-3′). PCR cycling conditions were conducted in a total volume of 20 μl and containing 10 pmol of each primer and 2 μl of diluted cDNA template (800 ng cDNA). The thermal cycler conditions were composed of an initial step at 95°C for 5 min followed by 45 cycles of 95°C for 15 s, 60°C for 1 min and 72°C for 30 s and a step of 82°C for 5 s. To generate standard curves, the tenfold serial dilutions covering a range of the eluted cDNA was considered as a template in SYBR green qRT-PCR.

### Statistical analysis

Statistical analysis of *in vitro* experiment was done using GraphPad Prism version 4 (GraphPad Software, CA, USA). P-values were calculated by two-sided Student's *t*-test and the values below 0.05 were considered to be significant.

## Results

### PPI networks properties

A PPI network was constructed for each CRC stage based on the experimentally validated PPI interactions. The PPI networks included 214 nodes and 169 edges (for PPI network of S_I_), 179 nodes and 219 edges (for PPI network of S_II_), 196 nodes and 152 edges (for PPI network of S_III_), and 203 nodes and 146 edges (for PPI network of S_IV_). A decreasing tendency of the distribution of node degree implicated a biological scale-free pattern of four constructed PPI networks (data not shown). The main subnetworks were extracted from each PPI network which is illustrated in Supplementary Figure 1. *CDK1*, *UBC*, *ESR1* and *ATXN1* genes had the highest degree of connectivity in the main subnetworks of S_I_, S_II_, S_III_ and S_IV_, respectively.

### Hub genes of each PPI network

Topological analysis of the PPI networks revealed several hub genes as major regulators of CRC initiation, progression and invasion. The results of all topological measures had a relatively high overlap. Interestingly, many of cell cycle-related genes were identified as hub genes in their corresponding PPI networks. The top ten hub genes derived from interpretation of each PPI network are listed in Table 1.

**Table 1.** **Top 10 hub genes identified from analysis of each protein–protein interaction network.**

**NumUndE**	**D**	**BetCen**	**CluCoe**	**CloCen**	**E**	**NeiCon**	**TopCoe**	**Avg.ShoPat**
**Hub genes of S_I_ of CRC**								

CDK1	CDK1	PALB2	CKS2	NUP37	PPAT	FBXO5	NUP37	CBX3

NPM1	NPM1	CDK1	NIFK	NUP160	PAICS	TOP2A	NUP160	EXOSC3

CCNB1	CCNB1	CSE1L	SKP2	NUP107	DIMT1	PTTG1	NUP107	EEF1E1

SNRPD2	SNRPD2	GNL3	CENPE	PALB2	POLR1B	KIF20B	SKP2	HELLS

BUB1	BUB1	BCCIP	NUP37	CD44	RPP40	CEP55	SPC25	HSPE1

CKS1B	CKS1B	NOP58	NUP160	MAD2L1	HELLS	DDX21	CENPE	RPP40

DKC1	DKC1	NPM1	DTL	PPARD	EEF1E1	CPSF3	DTL	POLR1B

RFC4	GART	DKC1	NUP107	NCAPG2	EXOSC3	NUDCD1	NIFK	DIMT1

RFC5	KIF11	KPNA2	SPC25	SMC2	CBX3	CDC25C	NUF2	EXOSC8

DTL	EXOSC3	CCNB1	PPP40	NUF2	KIF20B	NUP107	CDK1	NPM1

**Hub genes of S_II_ of CRC**								

UBC	UBC	UBC	CEBPG	UBC	SORBS1	DNAJC5	SORBS1	SLC35D1

YWHAB	YWHAB	AURKA	ATXN3	YWHAB	WIF1	JAG1	ZFP36L2	ZNF713

AURKA	AURKA	DNAJB12	NCOA6	AURKA	KLHL29	SLC25A23	PARD6B	MBTPS2

SNRNP200	SNRNP200	YWHAB	CASK	SNRNP200	YWHAB	PROCR	AAR2	SYK

CEBPB	CEBPB	EIF6	TPX2	EIF4E2	AURKA	G2E3	TPX2	PXMP4

AMOTL2	AMOTL2	SNRNP200	TLK2	ARAF	SNRNP200	TRERF1	NUF2	DYNLRB1

TOP1	COPS6	EIF4E2	PDHA1	CDC25C	EIF4E2	PIGU	TLK2	TMEM189

ARAF	ARAF	CEBPB	CTNNBL1	TNFAIP3	ARAF	ZNF687	MAPRE1	RALGAPB

COPS6	TOP1	AMOTL2	HERC5	CEBPB	CDC25C	DUSP1	CEBPG	ARFGAP1

EIF4EB	CDC25C	TPX2	CEBPB	CASK	TOP1	AURKA	PDHA1	ZNF687

**Hub genes of S_III_ of CRC**								

CABLES2	ESR1	TRIM23	HIP1	TRIM23	HIP1	RPS12	HIP1	CABLES2

PCK1	ARRB1	ESR1	ITSN1	INTS9	ITSN1	AKT2	ITSN1	PCK1

MGA	RYBP	ARRB1	YY1	TGFB1	EPN1	PAK1	MED23	MGA

CDK3	YY1	YY1	E2F2	INTS10	PCK1	IRS1	FASN	CDK3

EPN1	CDK3	AP2B1	RYBP	ITGB8	CABLES2	EPN1	RYBP	EPN1

HIP1	HIP1	MED23	AP2B1	ESR1	AP2B1	PTAFR	CDK3	HIP1

ITSN1	ITSN1	E2F2	TRIM23	DMRT3	FASN	YY1	E2F2	ITSN1

PTAFR	FASN	CDK3	ESR1	CCDC25	PTAFR	HIP1	YY1	PTAFR

FASN	AP2B1	FASN	ARRB1	MED23	CDK3	ITSN1	AP2B1	FASN

AP2B1	E2F2	HIP1	EPN1	HIP1	ESR1	AP2B1	ESR1	TRIM23

**Hub genes of S_IV_ of CRC**								

ATXN1	ATXN1	MAP4	ATXN1	UBXN1	SNX2	LDB1	HIVEP1	SCAMP1

SMAD2	SMAD2	ATXN1	SMAD2	ARHGEF12	PTPRK	WBSCR16	FAM193B	SMG9

TRIM23	TRIM23	SMAD2	TRIM23	SF3B6	CREB1	PUM1	TRIP6	FAM208B

INSR	INSR	FAM193B	INSR	AKT2	SCAMP1	LRSAM1	FAM208B	SNX2

YY1	YY1	TRIM23	YY1	ANKRD28	INSR	HIVEP1	YY1	PTPRK

TRIP6	TRIP6	INSR	FAM208B	PHLDA3	YY1	FAM193B	INSR	CREB1

FAM208B	FAM208B	YY1	TRIP6	MAP4	RUNX1	RUNX1	TRIM23	AFAP1L1

SCAMP1	SCAMP1	TRIP6	SCAMP1	TAF9	TNRC6B	TNRC6B	SMAD2	TRIM23

UBXN1	UBXN1	FAM208B	SMG9	ARHGEF2	AFAP1L1	TRIP6	ATXN1	RUNX1

SMG9	SMG9	UBXN1	UBXN1	YY1	ATXN1	SCAMP1	MAP4	PTPRK

Avg.ShoPat: Average shortest path length; BetCen: Betweenness centrality; CloCen: Closeness centrality; CluCoe: Clustering coefficient;  D: Degree; E: Eccentricity, NeiCon: Neighborhood connectivity; NumUndE: Number of undirected edge; TopCoe: Topological coefficient.

### Cell cycle genes are remarkably involved in CRC initiation & progression

**Figure 1.**
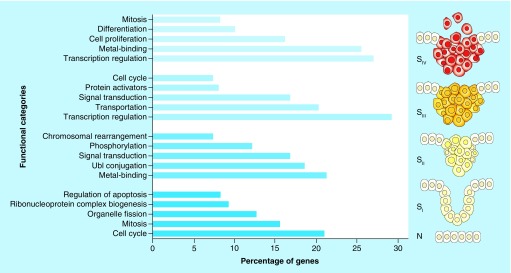
**Top five functional categories (categories with p-value < 0.05 containing at least 10 genes) that significantly enriched for each DEG list.** The right schematic representation of colon cancer progression is based on a model from Fearon and Vogelstein [26]. DEG: Differentially expressed gene; N: Normal stage; S_I_: Stage 1; S_II: Stage 2;_ S_III: Stage 3;_ S_IV: Stage 4_.

The results of GO demonstrated that, except for S_II_, the cell cycle pathway is remarkably involved in CRC initiation as well as its progression. The UBI–proteasome pathway, an intracellular process involved in protein degradation, was identified as the most significant enriched term for the PPI network which made from the DEGs of S_II_. Moreover, signal transduction seems to be critical in the regulation of gene expression in S_II_ and S_III_ of CRC (Figure 1).

**Figure 2.**
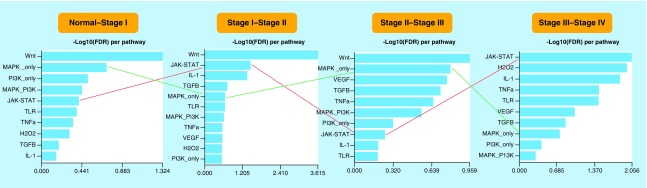
**Signaling pathway enrichment of the DEGs of four CRC stages.** MAPK signaling pathway (green line) and JAK-STAT signaling pathway (red line) had a negative correlation during colorectal cancer progression. Every significant hit in SPEED had an FDR <0.05. DEG: Differentially expressed gene; CRC: Colorectal cancer; FDR: False discovery rate.

Signaling pathway enrichment was carried out utilizing SPEED and Kyoto Encyclopedia of Genes and Genomes tools. In this step, only up-regulated DEGs were considered for pathway enrichment analyses to decipher activated signaling pathways during CRC progression. The results showed that the Wnt signaling pathway was the most significant signaling pathway in S_I_-S_III_ implying the indispensable role in initiation and progression of CRC from S_I_ to S_III_. However, this signaling pathway was not significantly enriched in S_IV_ suggesting that the metastasis of CRC is not considerably dependent on Wnt signaling pathway activity. The MAPK signaling pathway was shown to be especially active in S_I_ and S_III_. In contrast, the JAK-STAT signaling pathway was substantially enriched in the DEGs of S_II_ and S_IV_ (Figure 2).

### DEG-GO network construction for distant metastasis stage of CRC

**Figure 3.**
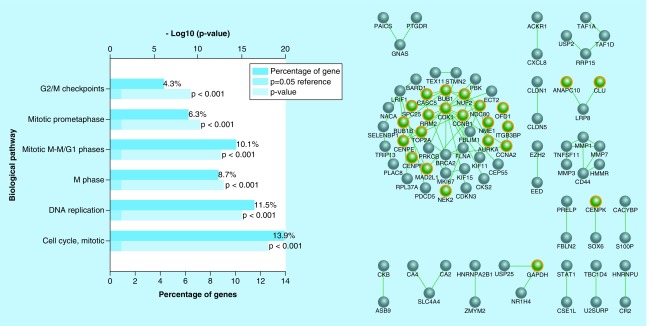
**Gene enrichment and network analysis of CRC's S_IV_ gene signature.** **(Left)** GO of the DEGs obtained from gene expression analysis of CRC's S_IV_ compared to adjacent normal tissue. **(Right)** Biological networks of these genes (green nodes) and their neighbors (gray nodes). GO: Gene ontology; DEG: Differentially expressed gene; CRC: Colorectal cancer; S_IV_: Stage 4

The DEGs of S_IV_ were obtained from analysis of gene expression profile of this stage compared with the adjacent normal cells. The up-regulated DEGs with fold change larger than 1 and p-value smaller than 0.05 were selected for GO studies. The results of GO revealed that the cell cycle related pathways are actively involved in CRC invasion. Furthermore, gene regulatory of these DEGs with their neighbors remarkably contributed to cell proliferation events. (Figure 3).

**Figure 4.**
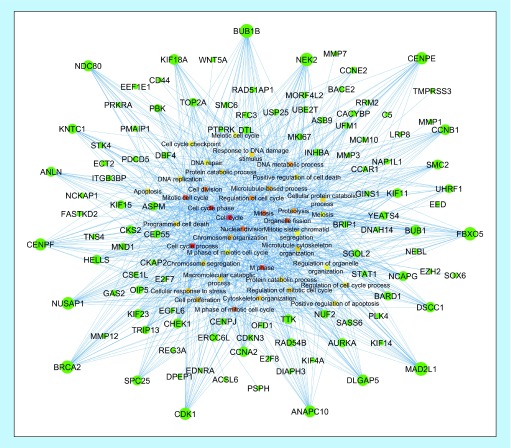
**DEG-GO network constructed for up-regulated genes of CRC's S_IV_ when compared to its adjacent normal tissue.** Nodes (DEGs) with higher and lower out-degree measures are shown in bigger and smaller circles, respectively. Nodes (GO terms) with higher in-degree measures are indicated in dark colors, while nodes with lower in-degree measures are shown in bright colors. DEG: Differentially expressed gene; GO: Gene ontology; CRC: Colorectal cancer; SIV: Stage 4.

In order to identify the most important up-regulated DEGs involved in the aforementioned functional categories of CRC's S_IV_, a DEG-GO network was also reconstructed. Detailed interpretation of this network based on out-degree measure resulted in the identification of several hub genes such as *BUB1B*, *CENPE*, *CCNB1*, *FBXO5*, *MAD2L1*, *DLGP5*, *ANAPC10*, *CDK1*, *BRCA2*, *NUSAP1*, *CENPF*, *ANLN*, *NDC80* and *KIF18A* belong to the most significant enriched GO terms. The in-degree analysis of resultant DEG-GO network revealed several important intracellular molecular functions, particularly cell cycle associated pathways, as the most highly regulated pathways by the up-regulated DEGs of CRC's S_IV_ (Figure 4).

### The S_I_-S_IV_ of CRC share a high number of common genes

**Figure 5.**
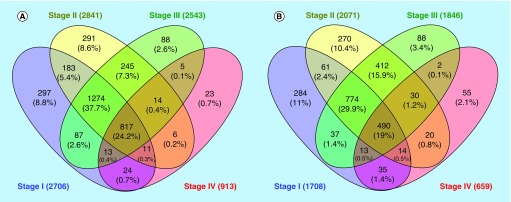
**Clustering of CRC stages according to their gene signatures.** Clustering of **(A)** up-regulated and **(B)** down-regulated DEGs resulted from preprocessing of CRC's S_I_-S_IV_ compared to their adjacent normal tissues. CRC: Colorectal cancer; DEG: Differentially expressed gene.

Clustering analysis of the DEGs obtained from each CRC stage compared to the respective normal tissues revealed that the four studied CRC stages (S_I_-S_IV_) share a notable number of overlapped genes involved in multiple cellular processes. Prior to clustering, up-regulated and down-regulated DEGs were determined and separated. The results of clustering identified 817 (24.2%) common up-regulated DEGs and 490 (19%) common down-regulated DEGs in all studied CRC stages. Nevertheless, the highest proportion of common genes was observed between S_I_, S_II_ and S_III_ (1274 genes among up-regulated DEGs and 774 genes among down-regulated DEGs) (Figure 5).

**Figure 6.**
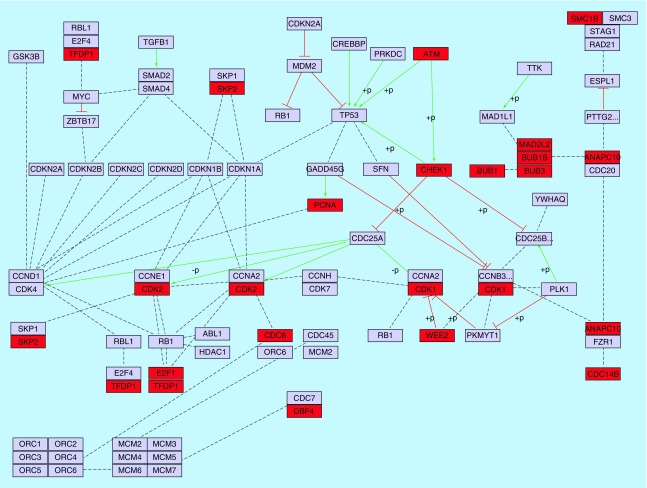
**Relationship between cell cycle and common up-regulated DEGs identified between S_I_-S_IV_ of CRC (red color).** Activation, inhibition and undirected interactions are shown with solid green lines, solid red lines and dotted black lines, respectively. The cell cycle pathway was extracted from KEGG database. DEG: Differentially expressed gene; S_I_-S_IV_: Stage 1 to Stage 4; CRC: Colorectal cancer; KEGG: Kyoto Encyclopedia of gene and genome.

Functional annotation of these common genes indicated that the majority of common up-regulated genes were actively involved in regulation of cell cycle (94 genes) and mitosis (57 genes). However, several other pathways such as regulation of RNA processing, DNA repair and protein metabolism were also significantly enriched. In contrast, the common down-regulated DEGs obviously participated in the regulation of apoptosis and intracellular signaling cascade (results not shown). Detailed analysis of these genes indicated that 21 genes (out of 94 genes) have a direct role in cell cycle pathway (Figure 6).

The main subnetwork was extracted from the PPI network constructed for common up-regulated DEGs and is depicted in Supplementary Figure 2. Calculation of several centrality measures such as Betweenness centrality and degree showed that *CAND1* gene is the most significant hub gene in the subnetwork. However, some other genes including *CDK2*, *EED*, *BRCA1*, *CDK1*, *BARD1*, *SRPK1*, *NPM1*, *PCNA*, *SRSF2*, *EZH2*, *HNRNPA1*, *HIST1H4C* and *HNRNPU* were also distinguished as important hub genes.

The enriched cellular functions for the SI-SIV common genes were examined. Our observations revealed that cell proliferation and apoptosis are the most significant functional categories enriched in common up-regulated and down-regulated genes, respectively. We further compared the expression levels of these genes during S_I_–S_IV_ of CRC. The results indicated that cell proliferation genes had the highest expression levels in S_I_ and S_IV_. However, apoptosis-related genes are down-regulated in S_III_ of CRC. *ANLN* and *CDK1* genes had the highest overexpression level in all CRC stages compared with adjacent normal cells, whereas *STAT1* was the most down-regulated gene in all CRC stages (Supplementary Figure 3).

### *ATXN1* &*CDK1* have significantly deregulated in metastatic SW620 cells

**Figure 7.**
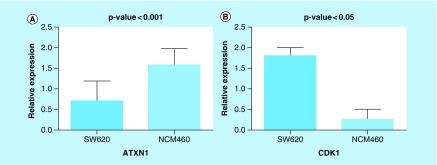
**Gene expression analysis using qRT-PCR.** Expression level of **(A)** *ATXN1* and **(B)** *CDK1* in SW620 and NCM460 cell lines. Changes in expression level were found to be significant (p < 0.05). qRT-PCR: Quantitative real-time PCR.

The expression levels of *ATXN1* and *CDK1* were normalized to the level of the *GAPDH* housekeeping gene which showed the most homogenous expression in both SW620 and NCM460 cell lines. Consistent with our bioinformatics analyses, the results of real-time PCR indicated that the expression of *ATXN1* (down-regulated, p-value < 0.001) and *CDK1* (up-regulated, p-value < 0.05) have significantly altered in metastatic SW620 cells compared with the normal colon NCM460 cells (Figure 7). This *in vitro* validation suggests that the introduced novel biomarkers of CRC can be reliably considered to be promising candidates for therapeutic applications.

## Discussion

Our study was aimed at modeling CRC initiation and invasion using deep analyses of microarray data. The results of this *in silico* study propose several potential candidates for the treatment of CRC as well as early diagnosis of this cancer. Consistent with previous studies, we found numerous important regulatory genes and transcription factors associated with CRC progression. These findings provide support for the efficiency of systems biology approaches in the identification of novel CRC biomarkers.

We found out that *CDK1*, *UBC*, *ESR1* and *ATXN1* are the most important hub genes of S_I_, S_II_, S_III_ and S_IV_ of CRC, respectively. Among these hub genes, *CDK1* and *UBC* were up-regulated in CRC samples compared with adjacent normal cells, while *ESR1* and *ATXN1* were down-regulated. *CDK1* is a member of Cyclin-dependent kinases (CDKs) family, which are serine/threonine kinases whose deregulation is associated with cancer progression. Hence, these cell cycle regulators have been targeted in a variety of human cancers [27]. Studies have shown that inhibition of *CDK1* enhances the sensitivity of CRC cells to anticancer compounds through caspase-dependent signaling pathways [28,29]. The *UBC*, also called second ubiquitin-conjugating enzyme or E2, is a member of ubiquitin-proteasome degradation system which contributes to the regulation of cell proliferation by mediating the selective degradation of target proteins. This enzyme also catalyzes a particular type of polyubiquitination, ‘Lys-63’-linked polyubiquitination, which does not result in protein degradation and leads to an enhanced cell survival through regulating cell cycle and DNA repair pathways [30,31]. Therefore, up-regulation of *UBC* may enhance the survival of CRC cell through maintaining the cell cycle and DNA repair abilities of the cell. *ESR1* is a nuclear hormone receptor that encodes estrogen receptor α involved in the regulation eukaryotic cell proliferation and differentiation. Although, *ESR1* is frequently amplified in breast cancer cells [32], methylation-dependent silencing of this gene by CGI (CpG island) has been previously reported [33]. CGI regions are unmethylated short and dispersed sequences of DNA with a high frequency of CpG dinucleotides which are highly associated with the bulk genome. Furthermore, evaluation of the *ESR1* methylation status uncovered that significant methylation of this gene occurred in patients with CRC and is associated with markedly diminished *ESR1* expression in colorectal tumors examined [34]. This suggests the possibility of tumor suppressor activity for *ESR1* in CRC. Accordingly, estrogens might reduce CRC development via preventing the DNA methylation of *ESR1* [35]. *ATXN1* is a chromatin-binding factor that functions as a corepressor involved in transcriptional repression by directly repressing *CBF1*, a transcription factor that is crucial for the Notch signaling pathway [36]. It is now widely accepted that Notch pathway is substantially required for initiation of CRC as well as maintenance and self-renewal of CRC initiating cells [37]. Furthermore, Sonoshita *et al*. found that activation of *DAB1/Dab1*, a Notch signaling target gene, can promote CRC invasion and metastasis [38]. Kim and colleagues have found a negative crosstalk between Notch and Wnt/β-catenin signaling pathways [39]. Interestingly, the signaling pathway enrichment revealed that Wnt signaling is actively involved in regulation of S_I_, S_II_ and S_III_ of CRC, but the activity of this signaling pathway has significantly diminished during S_IV_.

Wnt signaling pathway was found to be a key player in the initiation and progression on CRC, but not in metastasis stage. It is now well understood that Wnt signaling is a critical event for initiating the genesis of most CRCs, especially through loss of its negative regulator, Adenomatous Polyposis Coli [40]. Moreover, both β-catenin-dependent and β-catenin-independent Wnt signaling enhance CRC progression via regulating epithelial–mesenchymal transition [41]. For decades, components of this signaling pathway served as interesting targets in human CRC. In addition to the importance of Wnt signaling pathway, we found a negative correlation between JAK-STAT and MAPK signaling pathways during CRC progression suggesting a dual regulatory role of JAK-STAT/MAPK cascades in CRC progression and invasion. Wu and colleagues reported that cooperation of PAR2/MAPKs/NF-κB signal transduction pathways promotes proliferation and migration of colon cancer cell line SW620 [42]. The crosstalk between these signaling pathways is complex and each pathway can regulate the activation of the other at multiple levels. Consistent with this, Krasilnikov and colleagues have found that PI3K and MAPK signaling down-regulate JAK-STAT signaling in human melanoma cells via inhibiting tyrosine phosphorylation of JAK/STATs [43]. However, *STAT1* was identified as the most down-regulated gene in all CRC stages. In our previous study, this gene was also found to be down-regulated in metastatic CRC samples compared with the nonmetastatic samples. Modular analyses of CRC metastasis-derived PPI network corroborated that *STAT1* functions in one of the most important protein complexes correlated with CRC metastasis [7]. Considering the role of MAPK and JAK-STAT signaling pathways in the regulation of cell proliferation, we suggest that down-regulation of each pathway is recouped by activation of the other pathway. This mechanism of regulation may be indispensable for providing the favorable microenvironment for CRC development [44,45].

Interestingly, the clustering analyses demonstrated a remarkable overlap between all studied CRC stages. Functional annotation of common DEGs revealed that majority of common up- and down-regulated genes are obviously contributed to the regulation of cell proliferation and apoptosis, respectively. *CAND1*, *CDK2*, *EED*, *BRCA1* and *BARD1* were identified as the most important hub genes up-regulated in all CRC stages. Several evidence have indicated the disruption of *CAND1* in prostate cancer [46]. Nevertheless, the function of this gene in CRC has poorly been investigated. *CDK2* is a serine/threonine kinase which its activity is dispensable for controlling cell cycle and mitosis [47]. Inhibition of *CDK2* in CRC is substantially associated with enhanced cell death and suppressed cell proliferation implying its crucial function in CRC development [48,49]. Consistent with our results, *EED* and *BARD1* have been proposed as promising prognostic markers in CRC and their expression correlate with CRC progression and aggressive clinical behavior [50,51]. *BARD1* is an E3 ubiquitin-protein ligase which acts as a heterodimer with *BRCA1* (*BRCA1*-*BARD1*). This protein heterodimer mediates the formation of ‘Lys-6’-linked polyubiquitin chains, and subsequently regulates a wide range of intracellular pathways such as DNA repair, regulation of protein degradation, transcription and genomic stability [52,53].

## Conclusion

Our findings suggest a dynamic model of CRC initiation, progression and invasion. The results were highly compatible with previous studies. Moreover, several novel biomarkers were also proposed for each stage of CRC. The cell cycle and signal transduction genes were apparently involved in regulation of CRC stages. Moreover, Wnt, MAPK and JAK-STAT signaling pathways seemed to be crucial in the regulation of CRC stages. However, the results of this study need to be investigated in greater details by further experimental methods.

## Future perspective

Despite decades of investigations, clinicians are still faced with numerous challenges in the diagnosis and treatment of patients with a specific stage of CRC. This might be due to deregulation of particular gene signatures in each CRC stage. Thus, identification of drug targets based on molecular mechanisms of each CRC stage can be very helpful in targeted therapy of CRC. In this study, we separately analyzed gene expression profiles of each CRC stage (i.e., S_I_-S_IV_) and integrated the results of gene expression analysis with PPI networks. Several genes and regulatory networks were found in each CRC stage. Cell cycle and signal transduction genes showed critical roles in early stages of CRC. Intriguingly, a crosstalk between MAPK and JAK-STAT signaling pathways during CRC progression was also predicted *in silico*. Although, a part of *in silico* findings was validated *in vitro*, there is a need to further validate this study's results. Moreover, parallel analysis of other datasets (e.g., microarray or next generation sequencing data) would increase the reliability of these predictions.

**Summary points**

**Background**

- Rapid and robust screening for biomarkers in each stage of colorectal cancer (CRC) may result in early diagnosis and reliable treatment programs.
- Computational integration of gene expression profiles with protein–protein interaction networks have shown robustness in identifying key target genes and regulatory networks in human cancers.

**Experiment**

- Microarray dataset GSE21510 was obtained from gene expression omnibus database and analyzed with GEO2R.
- Cytoscape and FunRich packages were used for construction and analyses of protein–protein interaction networks.
- BiNGO, SPEED and DAVID tools were used for functional enrichment of differentially expressed genes.

**Results & discussion**

- *CDK1*, *UBC*, *ESR1* and *ATXN1* genes were detected as the most significant hub genes of S_I_, S_II_, S_III_ and S_IV_ of CRC, respectively.
- Cell cycle genes were highly involved in S_I_-S_III_ of CRC suggesting their crucial role in CRC initiation and progression.
- Wnt signaling pathway was particularly enriched in S_I_-S_III_, but not in S_IV_. Whereas, MAPK and JAK-STAT signaling pathways had a negative correlation during CRC stages.
- A high number of common genes were identified between S_I_, S_II_ and S_III_ implying their close biological properties.
- Some *in silico* findings were validated *in vitro* implicating that the results of this study can be used in CRC diagnosis and prevention programs.
