# Supplementary material for: Key genes and regulatory networks involved in the initiation, progression and invasion of colorectal cancer
Source: Future Sci OA. 2018 Jan 24;4(3):FSO278. doi: 10.4155/fsoa-2017-0108 (PMC5859335; doi:10.4155/fsoa-2017-0108)
Supplement: Supplementary file 1 [file fsoa-04-278-s1.docx]

**Title: Key genes and regulatory networks involved in the initiation, progression and invasion of colorectal cancer**

**Running title: Novel biomarkers of CRC stages**

**Matin Asghari^1†^, Mohammad Foad Abazari^2†^, Hanieh Bokharaie^3^, Maryam Nouri Aleagha^2^, Vahdat Poortahmasebi^4^, Hassan Askari^5^, Sepehr Torabinejad^2^, Abbas Ardalan^6^, Navid Negaresh^7^, Atousa Ataie^8^, Parisa Pazooki^9^, Mansour Poorebrahim^10🖂^**

1. Department of Molecular Biotechnology, Cell Science Research Center, Royan Institute of Biotechnology, ACECR, Isfahan, Iran.
2. Department of Genetics, Islamic Azad University, Tehran Medical Branch, Tehran, Iran
3. Department of Genetics, Faculty of Basic Sciences, Science and Research Branch, Azad University, Tehran, Iran.
4. Hepatitis B Molecular Laboratory, Department of Virology, School of Public Health, Tehran University of Medical Sciences, Tehran, Iran
5. Department of Physiology, Faculty of Medicine, Tehran University of Medical Sciences, Tehran, Iran
6. Department of Biology, Faculty of Sciences, Arak University, Arak, Iran
7. Department of Medicine, Faculty of Medicine, Qom Branch, Islamic Azad University, Qom, Iran
8. Institute of Fundamental Medicine and Biology, Kazan Federal University, Kazan, Russia
9. Department of biological sciences, Tehran north branch, Islamic Azad university, Tehran, Iran
10. Department of Medical Biotechnology, School of Advanced Technologies in Medicine, Tehran University of Medical Sciences, Tehran, Iran

**† Authors contributed equally**

**🖂 Corresponding author:** Mansour Poorebrahim, Department of Medical Biotechnology, School of Advanced Technologies in Medicine, Tehran University of Medical Sciences, Tehran, Iran. E-mail: [mpoorebrahim@razi.tums.ac.ir](mailto:mpoorebrahim@razi.tums.ac.ir)


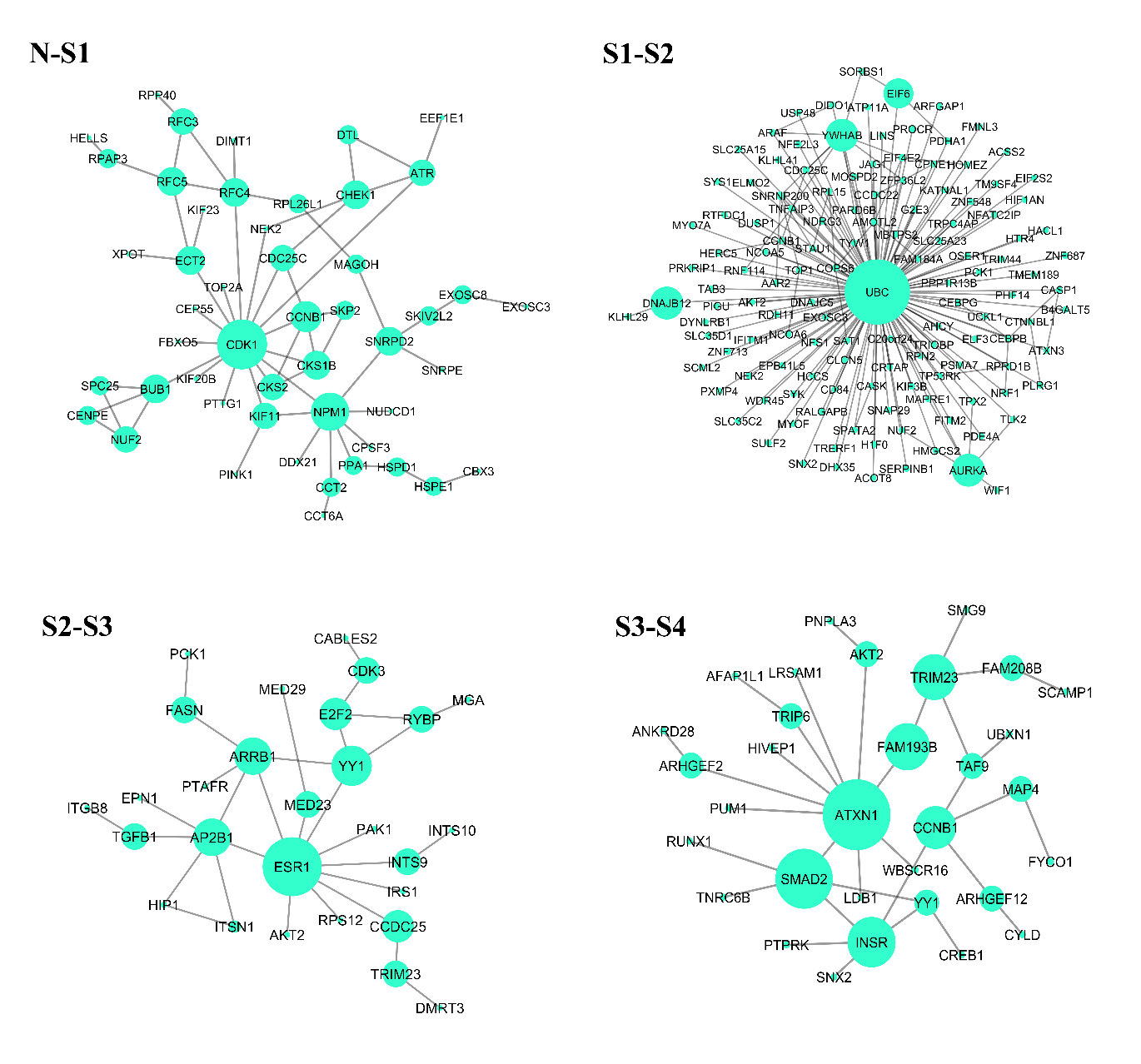


**Supplementary Figure 1**. The main sub-network extracted from each PPI network. Nodes with higher degree measures are represented in larger circles. N: normal stage, S1: stage I, S2: stage II, S3, stage III and S4: stage IV.


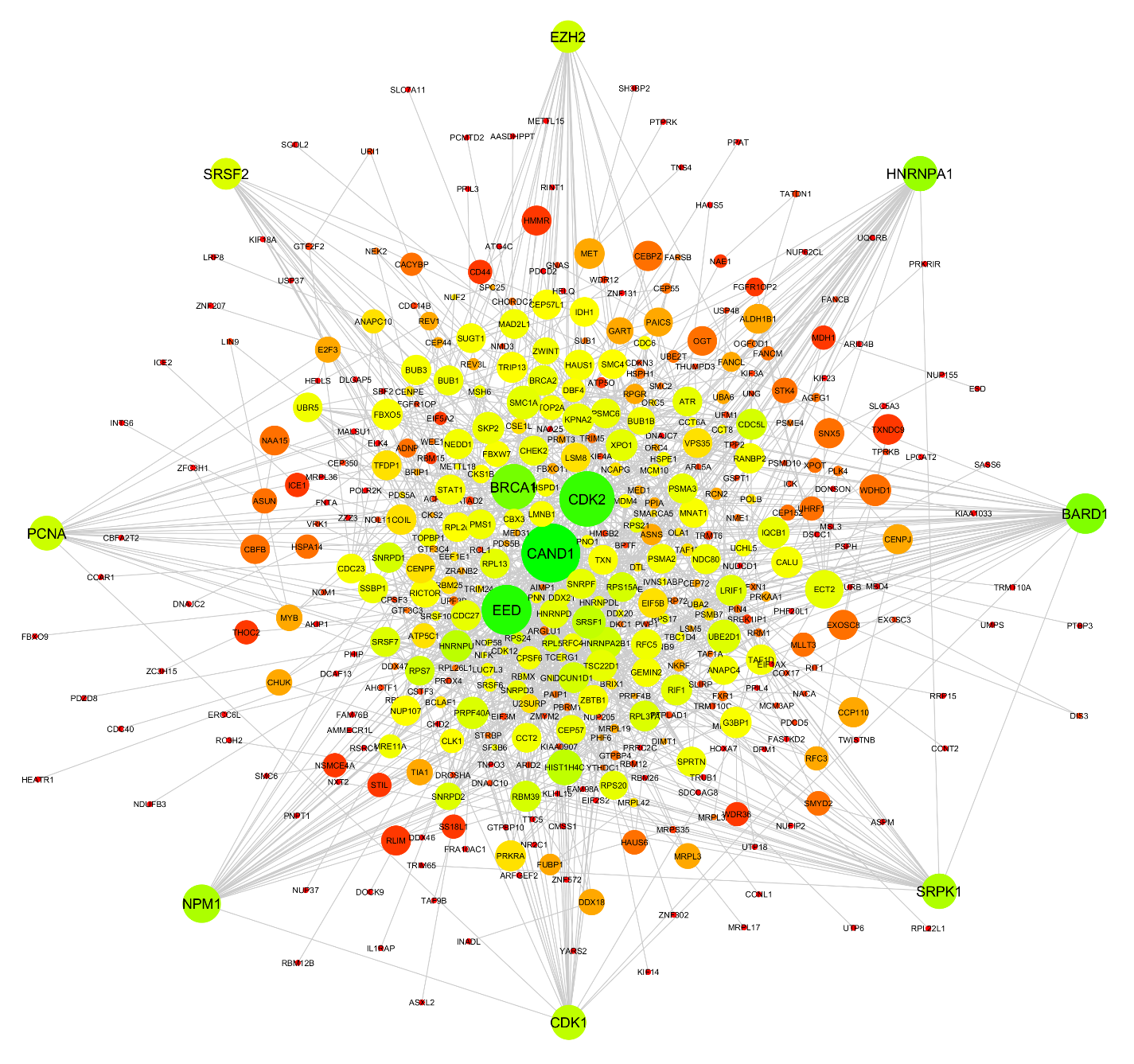


**Supplementary Figure 2**. The main sub-network of PPI network of common up-regulated genes. Nodes with higher and lower Betweenness measures are represented in bigger and smaller circles, respectively. Nodes with low degree measures have represented in dark colors, while nodes with higher degree measures are represented in bright colors.


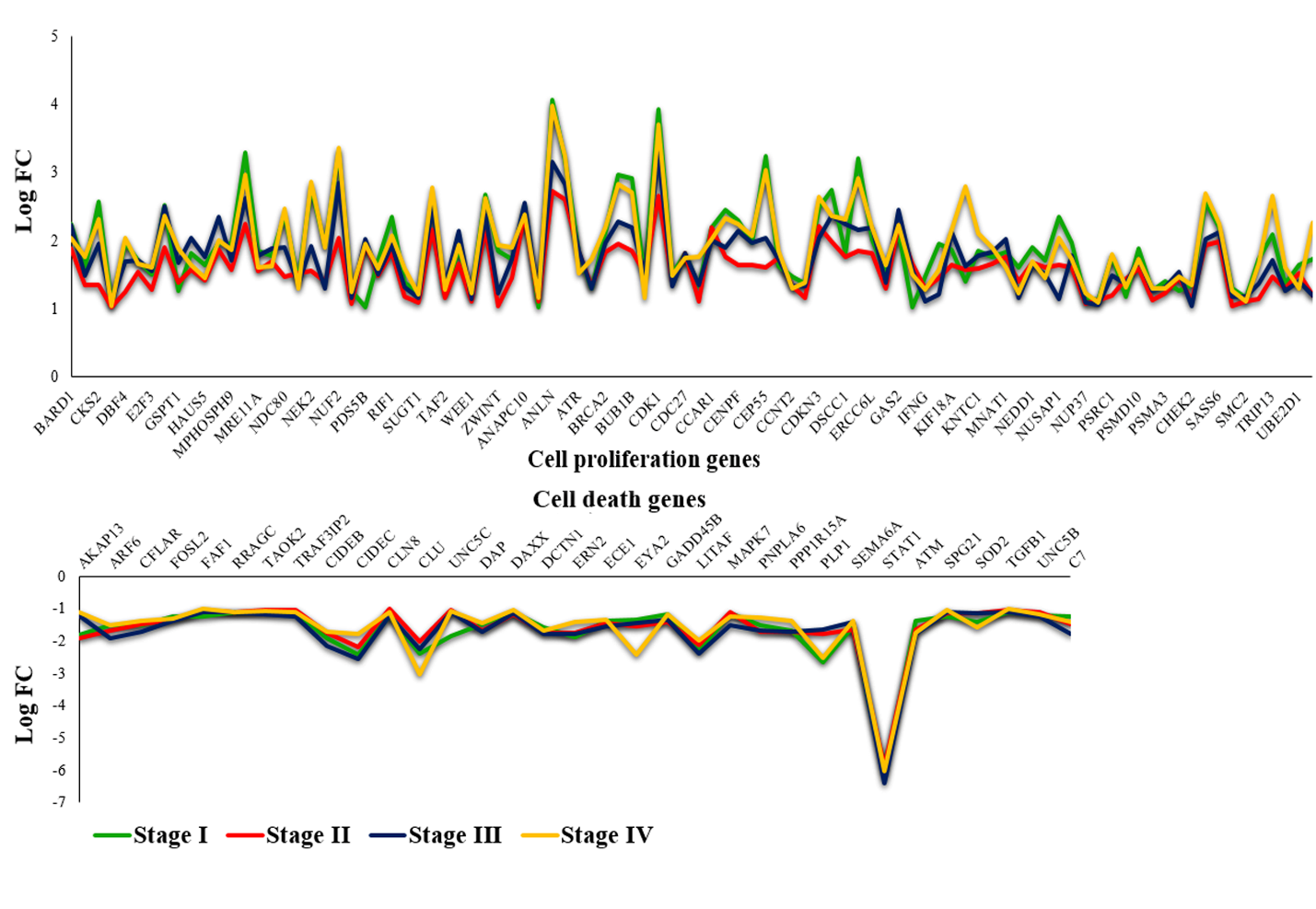


**Supplementary Figure 3**. Expression levels of cell proliferation genes in common up-regulated DEGs (up) and apoptosis genes in common down-regulated DEGs (down) of S_I_-S_IV_ of CRC. Stages I, II, III and IV of CRC are shown in green, red, blue and yellow colors, respectively.
